# Utility of the visual system to monitor neurodegeneration in multiple sclerosis

**DOI:** 10.3389/fnmol.2023.1125115

**Published:** 2023-03-29

**Authors:** Gabrielle M. Mey, Tara M. DeSilva

**Affiliations:** Department of Neurosciences, Cleveland Clinic, Cleveland, OH, United States

**Keywords:** neurodegeneration, demyelination, retina, optic nerve, neuroinflammation

## Abstract

Neurodegeneration occurs early in the multiple sclerosis (MS) disease course and is an important driver of permanent disability. Current immunomodulatory therapies do not directly target neuronal health; thus, there is a critical need to develop neuroprotective strategies in MS. Outcome measures in clinical trials primarily evaluate disease activity and clinical disability scores rather than measures of neurodegeneration. The visual system provides a noninvasive correlate of brain atrophy and neuronal function through structural and functional exams. Furthermore, optic nerve axons and their respective neuronal cell bodies in the retina, in addition to their synaptic input to the thalamus, provide a distinct anatomy to investigate neurodegenerative processes. This review discusses the utility of the visual system as an early output measure of neurodegeneration in MS as well as an important platform to evaluate neuroprotective strategies in preclinical models.

## 1. Introduction

Neurodegeneration in MS is indicative of permanent axonal and neuronal cell body loss leading ultimately to irreversible progressive decline. Immunomodulatory therapies to treat MS focus on preventing the proliferation, migration, and/or infiltration of peripheral immune cells into the central nervous system (CNS). While this can reduce inflammatory relapses that are characteristic of relapsing–remitting MS, evidence of subclinical inflammatory lesions on MRI still occurs (Fox and Cohen, 2001). Further, transected axons are observed in active MS lesions demonstrating that ongoing inflammation leads to axonal damage and contributes to long-term disease progression (Trapp et al., 1998). This suggests a critical need to provide protection to the axon itself, but there are currently no therapies for MS that are specifically neuroprotective. A better understanding of the pathogenesis underlying neurodegeneration in MS and more sensitive measures of its detection are necessary to improve and inform therapeutic strategies. The visual system has become an important and useful avenue to understand the relationship between demyelination, axonal injury, and neurodegeneration in the CNS. Visual system pathology is evident in both MS (Fisher et al., 2006; Green et al., 2010; Albrecht et al., 2012; Balk et al., 2015) and disease models (Shindler et al., 2006; Horstmann et al., 2013; Carbajal et al., 2015; Araújo et al., 2017; Jin et al., 2019), and visual assessments have been correlated with measures of neurodegeneration and disease progression. Certain visual tests are noninvasive and take little time to perform, making this system applicable to both research and clinical settings.

Visual tests offer insight into the extent of neurodegeneration, propensity for disease progression, and the efficacy of therapeutic intervention. Optical coherence tomography (OCT) in particular is a noninvasive test that takes only minutes to perform and allows the integrity of the retina and the thicknesses of its distinct cellular layers to be measured (Huang et al., 1991). Degeneration of the retina measured by OCT is associated with brain atrophy and thalamic volume in MS (Gordon-Lipkin et al., 2007; Saidha et al., 2015; Mey et al., 2022a), and with loss of retinal ganglion cells (RGCs) in experimental autoimmune encephalomyelitis (EAE; Cruz-Herranz et al., 2019). Additionally, the retinal vasculature can be evaluated in a specialized form of OCT with angiography (OCT-A), which allows the visualization and measurement of vascular densities and distribution (Wang et al., 2018). Decreases in retinal vascular plexus densities are observed in MS and are associated with MS clinical disability and visual acuity, suggesting its utility as a marker for worsening disease (Lanzillo et al., 2018; Murphy et al., 2020). Vascular changes have implications for hypoxia, hypo-perfusion, and energy imbalances that can lead to mitochondrial dysfunction and neurodegeneration (Kleerekooper et al., 2020). Therefore, OCT and OCT-A provide early information for structural changes that may indicate risk for degeneration.

Aside from structural changes in the retina, visual functional tests assess alterations in neuronal function. Electroretinograms (ERG) can be utilized to determine the functional health of the retina in MS (You et al., 2018). One particular form of this test, pattern ERG, is able to provide information specifically for RGCs (Holder, 1997; Bach et al., 2018), which is important for understanding optic nerve injury (RGC axons) relative to neurodegeneration. Recording visual evoked potentials (VEP) is one method to quantify neuronal responses to visual stimuli, with delayed VEP latency associated with demyelination, often of the optic nerve (Halliday et al., 1972; You et al., 2011). Another measure of visual function is high- and low-contrast visual acuity, which is associated with retinal degeneration and clinical disability in MS (reviewed in Balcer et al., 2017). Finally, motion perception deficits have been studied as a potential marker of axonal degeneration and demyelination in MS (Ayadi et al., 2021). Decreases in motion perception have been associated with retinal thinning, visual acuity, and VEP latency (Backner et al., 2019; Ayadi et al., 2021). Here, we discuss current literature on visual assessments relevant for monitoring MS, focusing on the potential for earlier detection of neurodegeneration.

## 2. Monitoring MS through the visual system

### 2.1. OCT and OCT-A are correlates of CNS pathology in MS

While magnetic resonance imaging (MRI) and neuroperformance assessments are the standard of care to monitor MS (Thompson et al., 2018), visual assessments can aid in earlier detection of neurodegeneration. Thinning of the inner retinal layers (retinal nerve fiber layer—RNFL and ganglion cell/inner plexiform layer—GCIPL) measured by OCT are associated with brain atrophy and loss of white matter integrity regardless of whether a patient previously had optic neuritis, with greater rates of retinal degeneration observed in relapsing–remitting MS (RRMS; Ratchford et al., 2013; Saidha et al., 2015; Alves et al., 2018; Paul et al., 2021). These studies suggest that retinal degeneration reflects CNS atrophy and emphasizes the need for neuroprotective therapies early in the disease course. Currently, thalamic atrophy is one of the strongest predictors of MS disease worsening (Eshaghi et al., 2018; Magon et al., 2020). We recently published that retinal thickness measured by OCT correlates with thalamic volume in regions receiving afferent input from white matter tracts during RRMS (Mey et al., 2022a), providing an early measure of neurodegeneration. In progressive MS, rates of retinal thinning were found to be faster than in RRMS regardless of age or of disease-modifying therapy (Sotirchos et al., 2020). These studies suggest that retinal thickness may be an important marker for progressive MS and a global indicator for neurodegeneration in the CNS.

Measuring vascular changes in the retina using OCT-A is an emerging technology to monitor changes in MS. Decreased retinal vascular densities in patients with MS with and without a history of optic neuritis were associated with Expanded Disability Status Score (EDSS), visual acuity, and neurological performance (Lanzillo et al., 2018; Murphy et al., 2020). Another study by Jiang et al. (2020) showed that retinal vascular density was positively associated with EDSS, but noted that an *inecrease* in retinal vascular density was associated with a *decrease* in low-contrast letter acuity in RRMS. Higher density of choriocapillaris in the retina was also associated with previous inflammatory activity in MS (Feucht et al., 2019). These studies suggest that vascular alterations may be informative for both disease severity and ongoing inflammatory activity. Additionally, OCT-A may aid in the diagnosis of MS vs. neuromyelitis optica spectrum disorders, with important implications for appropriate therapeutic intervention (Rogaczewska et al., 2021; Tiftikcioglu et al., 2022). Taken together, these studies warrant further exploration into the usefulness of OCT-A as a predictor of MS disease progression and neurodegeneration in conjunction with OCT.

### 2.2. Functional visual changes reflect neurodegeneration and demyelination

Visual dysfunction is one of the most common symptoms in MS, with abnormalities occurring along both afferent and efferent tracts (Graves and Balcer, 2010). Visual acuity, particularly low-contrast acuity, is an outcome measure of neuroperformance that correlates with retinal thinning measured by OCT and EDSS measures of MS disability (Fisher et al., 2006; Talman et al., 2010; Schinzel et al., 2014; Murphy et al., 2020). Low-contrast visual acuity correlates with OCT measures of RNFL (unmyelinated RGC axons) and GCL (RGC cell bodies), providing a functional correlate for structural changes in the retina in MS (Walter et al., 2012). This supports the value of using visual acuity as a functional outcome measure when determining the efficacy of neuroprotective therapies in addition to measures of clinical disability and structural changes (i.e., neuronal loss in the retina; Fisher et al., 2006; Wu et al., 2007). ERG is a test of neuronal function in the retina where information from light is first transduced. A specific form of this test, pattern ERG, selectively measures the function of RGCs (Porciatti, 2015). Since it has been shown that RGCs degenerate subsequent to axonal loss in the optic nerve in the EAE preclinical model (Jin et al., 2019; Mey et al., 2022a), decreased RGC function may be indicative of neurodegeneration likely secondary to axonal injury.

VEPs provide information regarding demyelinating activity throughout the entire visual system from the optic nerve to the occipital cortex by measuring action potentials in response to visual stimulation. VEP latency, or the speed of nerve conduction, has been associated with demyelination (You et al., 2011), while a decrease in amplitude is associated with axonal injury or sustained demyelination leading to a decrease in nerve function even without a history of optic neuritis (Halliday et al., 1972; Jones and Brusa, 2003). This has particular relevance for early disease monitoring, as this indicator of demyelination and damage in the visual system assists in the diagnosis of clinically definite MS and detects optic nerve inflammatory activity early in disease course (Kallmann et al., 2006; Behbehani et al., 2017; Thompson et al., 2018). Of note, multifocal (mf) VEP (as opposed to full-field VEP) stimulates multiple small areas in the visual field, allowing for a more accurate detection of visual dysfunction as a consequence of demyelination (reviewed in Klistorner and Graham, 2021). Latency in mfVEP is increased especially in cases of optic neuritis (Fraser et al., 2006), but also in non-optic neuritis patients (Laron et al., 2010; Klistorner et al., 2021). Due to these beneficial characteristics, mfVEPs may be useful not only in detecting demyelinating activity and chronic demyelination, but also in evaluating remyelinating therapies (discussed in sections 3 and 4 below).

Measuring motion perception, another type of visual assessment, may predict both axonal loss and demyelination, though without regional specificity. A study by Ayadi et al. (2021) investigated two cohorts of people with MS in Berlin and Sydney and found that motion perception deficits correlated with RNFL and GCIPL thicknesses and visual acuity. This suggests that motion perception may be correlated with axonal loss. However, in the cohort from Sydney, where patients had more pronounced optic neuritis, motion perception also correlated with mfVEP latencies (Ayadi et al., 2021). This is consistent with a study showing that, especially in patients with a history of optic neuritis, full-field VEP latencies were inversely associated with motion perception ability (Backner et al., 2019). Motion perception may therefore be a promising indicator for significant demyelination and axonal degeneration, suggesting an important relevance for early disease monitoring and potential for assessing remyelination.

Overall, these visual tests have clinical relevance for axonal and neuronal changes that occur in MS that reflect CNS pathology and atrophy. While this is critical for monitoring disease activity and risk for progression and/or recovery in MS, there is still a need to understand the basic cellular pathology underlying visual changes. Therefore, histological studies and preclinical rodent models have been used to delve into visual system pathology and how it relates to neurodegeneration and progression in MS.

## 3. Investigating neurodegeneration in MS and disease models

### 3.1. MS histopathology in the CNS and visual system

**Figure 1:**
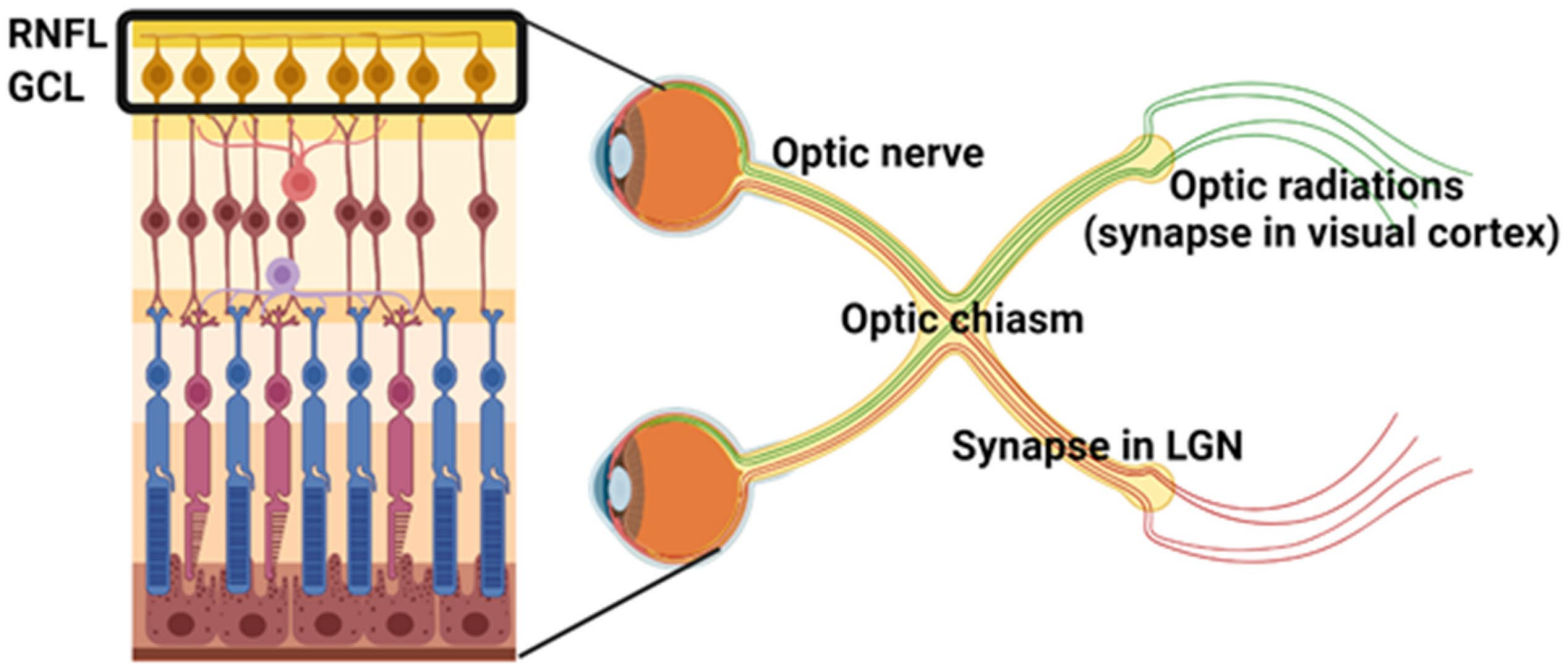
Organization of the anterior and posterior visual pathways. Retinal ganglion cells (RGCs) in the ganglion cell layer (GCL) are the innermost neurons of the retina. RGC axons comprise the unmyelinated retinal nerve fiber layer (RNFL) and become myelinated in the optic nerve. Optic nerve axons form presynaptic terminals in the lateral geniculate nucleus (LGN) of the thalamus. Together, this forms the anterior visual pathway. The afferent fibers from the dLGN emerge as the optic radiations and innervate V1 in the occipital region of the brain to process vision, forming the posterior visual pathway. Image created with BioRender.com.

The anatomy of the visual pathway facilitates a clear distinction between demyelination, axonal injury, and neurodegeneration in demyelinating disease. This is due to the organization of RGCs, their corresponding axons that are unmyelinated in the RNFL and become myelinated to form the optic nerve, and their associated synaptic connections in the dorsal lateral geniculate nucleus (dLGN) of the thalamus (Figure 1). Lesion formation in white matter tracts along the visual pathway and cell loss in the retina have been observed in the majority of postmortem MS tissue (Ikuta and Zimmerman, 1976; Toussaint et al., 1983; Green et al., 2010). Optic neuritis is a common early symptom of MS, with a 50% risk of developing MS within 15 years (The Optic Neuritis Study Group, 2008). However, thinning of the RNFL and GCL is observed regardless of a history of optic neuritis (Saidha et al., 2015; Graham et al., 2016). Research has therefore set out to investigate the pathology behind retinal thinning to corroborate structural and functional changes.

One important contributor to retinal thinning is trans-synaptic axonal degeneration, whereby damage in the posterior visual system (i.e., optic radiations and visual cortex, Figure 1) associates with RNFL thinning as well as global brain atrophy (Gabilondo et al., 2014; Balk et al., 2015). A decrease in LGN volume has also been associated with lesions in the optic radiations (Papadopoulou et al., 2019), which are axons from dLGN neurons that project to the occipital cortex (Figure 1). This is consistent with a study by Klistorner et al. (2022) showing that chronic demyelination drives axonal loss in the visual pathway, with mfVEP latency (measure of demyelination) associating with the rate of RNFL loss (measure of axonal loss). These studies indicate that retrograde and anterograde neurodegeneration occurs in the visual system following demyelinating activity in various white matter tracts and can be used to assess progressive axonal loss. However, there may also be independent mechanisms of degeneration that contribute to retinal thinning. In a large cohort of MS tissue, inflammation of the retina and abnormalities of retinal vasculature were observed across all MS disease courses (Green et al., 2010). These findings corroborate OCT-A abnormalities in patients with MS (Lanzillo et al., 2018; Murphy et al., 2020), suggesting that vascular dysregulation in MS is an important contributor to neurodegeneration.

Neuronal loss observed along the visual pathway in postmortem MS tissue confirms a spatial relationship between neurodegeneration and structural and functional changes observed with visual assessments. However, the temporal relationship between myelin/axonal injury and neurodegeneration is essential to help delineate mechanisms of neuronal loss and neuroprotection in the CNS. While associations can be made in clinical studies, preclinical models of demyelinating disease are crucial to investigate underlying mechanisms of neurodegeneration and potential methods of neuroprotection.

### 3.2. Visual pathology in animal models reflects CNS demyelination and degeneration

The most commonly used preclinical model for MS is the experimental autoimmune encephalomyelitis (EAE) model (Ransohoff, 2012). In C57BL/6 J mice, EAE recapitulates many of the T cell-mediated inflammatory responses that are observed in MS. This model also demonstrates retinal thinning by OCT, loss of RGCs, and functional visual deficits (Horstmann et al., 2013; Cruz-Herranz et al., 2019; Sekyi et al., 2021). We also recently established that the timeline of demyelination, consistent with delayed VEP, synaptic loss, and axonal injury relative to neurodegeneration in the visual system, mirrors that of the spinal cord where EAE has been typically studied (Mey et al., 2022a). Further, loss of RGCs has been correlated with EAE clinical scores, showing that neurodegeneration in the visual system is reflective of spinal cord pathology as evidenced by motor phenotype (Cruz-Herranz et al., 2019; Jin et al., 2019). These studies suggest that studying the visual system in EAE is a useful way to study mechanisms underlying neurodegeneration. EAE can also be used to study independent mechanisms of neurodegeneration other than secondary loss relative to demyelination. Cruz-Herranz et al. (2021) showed that in early EAE before clinical onset, subsets of microglia in the retina proliferate and express genes consistent with inflammatory pathways. Microglia density during early EAE in the retina was also associated with the degree of RGC loss at chronic EAE, suggesting that microglia may play a role in neuronal loss (Cruz-Herranz et al., 2021). Microglia have also been implicated in synaptic loss in the dLGN early in EAE, preceding optic nerve axonal loss, in a complement-mediated manner. Blocking complement signaling reduced microglial engulfment of synapses in the dLGN during early EAE and was accompanied by improved visual acuity (Werneburg et al., 2020). We have also shown that blocking a mediator of oligodendrocyte cell death in EAE, the AMPA type glutamate receptor, reduced demyelination and loss of myelinated axons in the spinal cord (Evonuk et al., 2020). This begs the question of how demyelination and axonal injury affect loss of synapses and neuronal function that has been investigated in other models of demyelination such as the cuprizone model.

Cuprizone is a copper chelator that induces oligodendrocyte cell death and demyelination followed by remyelination upon cuprizone diet cessation (Kipp et al., 2009). This model allows for the investigation of demyelination-induced axonal changes without the robust adaptive inflammatory responses that are observed in the EAE model. It has been shown that following cuprizone-induced demyelination, loss of excitatory synapses in the dLGN is accompanied by a shift toward inhibitory signaling (Araújo et al., 2017). VEP activity in the cuprizone model corresponds to demyelination and remyelination, indicating that VEP reflects demyelinating activity as it does in MS patients (Marenna et al., 2022). This also suggests that VEPs may reflect the efficacy of remyelinating therapy in preclinical models, providing utility in determining neuroprotective strategies in MS. A study by Crawford et al. (2009) showed that there is a limited window of time following demyelination in the cuprizone model in which neuronal function can be recovered. This emphasizes the importance of providing neuroprotection early in disease. The use of the visual system for early detection of structural and functional changes during MS is therefore critical to informing therapeutic strategies.

## 4. Evaluating therapeutic strategies and CNS repair

In terms of evaluating therapeutic efficacy, visual assessments with a functional purpose potentially have an important role for evaluating neuroprotective therapies. In preclinical models, VEP latency has been validated as a measure of demyelination and remyelination, making it useful to evaluate the efficacy of remyelinating therapies (Heidari et al., 2019; Cordano et al., 2022). In fact, VEPs have been used as an outcome measure in clinical trials to evaluate the safety and efficacy of potential remyelinating therapies clemastine fumarate (Green et al., 2017) and opicinumab (Cadavid et al., 2017). In a substudy of the opicinumab (RENEW) trial, mfVEP was used as a readout instead of full-field VEP, and showed that mfVEP may be a more sensitive way to assess remyelination in future studies (Klistorner et al., 2018). VEP or mfVEP as a functional readout has important clinical relevance as early intervention is key to preventing secondary neurodegeneration from sustained demyelination (reviewed in Mey et al. (2022b)). Low-contrast letter acuity has also been correlated with VEP, EDSS, and MRI activity (Balcer et al., 2017). Changes in visual acuity have predicted EDSS, making it an important functional readout of MS disability (Baier et al., 2005). This has implications both for detecting disease progression or delayed progression in response to therapeutic intervention. While structural visual assessments such as OCT cannot particularly evaluate repair (i.e., retinal thickness will not increase after neurons are already lost), a decrease in rate of retinal thinning can help indicate the efficacy of a disease-modifying or neuroprotective therapies. Overall, these studies indicate that the visual pathway serves as both a predictor of neurodegeneration as well as a measure of CNS recovery, providing a well-rounded system to evaluate MS progression and treatment.

## 5. Discussion

While MRI and clinical assessments remain the standard of care for MS (Thompson et al., 2018), additional assessments are critical for earlier disease diagnosis and detecting disease progression. Visual system pathology including retinal thinning, reduction of visual acuity, and delayed VEP, mfVEP, and ERG recordings are promising avenues to fill this void and provide helpful information to guide clinical care and therapeutic strategies (Saidha et al., 2015; Balcer et al., 2017; Graham and Klistorner, 2017). Animal models of disease have elucidated molecular mechanisms regarding how demyelination and neuroinflammation affect axonal integrity and neuronal function. Targeting these pathways has been shown to improve visual function and, in some cases, restore neuronal function following axonal damage (Crawford et al., 2009; Werneburg et al., 2020). OCT imaging, VEPs, and visual acuity are three particular assessments that have been used in both mice and humans, encouraging the translation of findings in preclinical models to the bedside. The studies highlighted in this review show the increasing impact of the visual system for early detection of neurodegeneration and to guide research toward improved care for MS.

## Author contributions

GM and TD conceptualized, wrote, and revised the original manuscript. GM generated Figure 1. All authors contributed to the article and approved the submitted version.

## Funding

This work was supported by the National Science Foundation (1648822), the National Eye Institute (R01EY025687, R01EY032342, P30EY025585, and T32 EY024236), and the National Multiple Sclerosis Society (FG-1807-31882 and FG-2108-38411).

## Conflict of interest

The authors declare that the research was conducted in the absence of any commercial or financial relationships that could be construed as a potential conflict of interest.

## Publisher’s note

All claims expressed in this article are solely those of the authors and do not necessarily represent those of their affiliated organizations, or those of the publisher, the editors and the reviewers. Any product that may be evaluated in this article, or claim that may be made by its manufacturer, is not guaranteed or endorsed by the publisher.

## References

[ref1] Albrecht P. Ringelstein M. Müller A. K. Keser N. Dietlein T. Lappas A.. (2012). Degeneration of retinal layers in multiple sclerosis subtypes quantified by optical coherence tomography. Mult. Scler. 18, 1422–1429. doi: 10.1177/1352458512439237, PMID: 22389411

[ref2] Alves C. Batista S. d'Almeida O. C. Sousa L. Cunha L. Bernardes R.. (2018). The retinal ganglion cell layer predicts normal-appearing white matter tract integrity in multiple sclerosis: a combined diffusion tensor imaging and optical coherence tomography approach. Hum. Brain Mapp. 39, 1712–1720. doi: 10.1002/hbm.23946, PMID: 29334156 PMC6866258

[ref3] Araújo S. E. S. Mendonça H. R. Wheeler N. A. Campello-Costa P. Jacobs K. M. Gomes F. C. A.. (2017). Inflammatory demyelination alters subcortical visual circuits. J. Neuroinflammation 14:162. doi: 10.1186/s12974-017-0936-0, PMID: 28821276 PMC5562979

[ref4] Ayadi N. Oertel F. C. Asseyer S. Rust R. Duchow A. Kuchling J.. (2021). Impaired motion perception is associated with functional and structural visual pathway damage in multiple sclerosis and neuromyelitis optica spectrum disorders. Mult. Scler. J. 28, 757–767. doi: 10.1177/13524585211032801, PMID: 34379018 PMC8978464

[ref5] Bach M. Cuno A. K. Hoffmann M. B. (2018). Retinal conduction speed analysis reveals different origins of the P50 and N95 components of the (multifocal) pattern electroretinogram. Exp. Eye Res. 169, 48–53. doi: 10.1016/j.exer.2018.01.021, PMID: 29374551

[ref6] Backner Y. Petrou P. Glick-Shames H. Raz N. Zimmermann H. Jost R.. (2019). Vision and vision-related measures in progressive multiple sclerosis [original research]. Front. Neurol. 10:455. doi: 10.3389/fneur.2019.00455, PMID: 31130910 PMC6509148

[ref7] Baier M. L. Cutter G. R. Rudick R. A. Miller D. Cohen J. A. Weinstock-Guttman B.. (2005). Low-contrast letter acuity testing captures visual dysfunction in patients with multiple sclerosis. Neurology 64, 992–995. doi: 10.1212/01.Wnl.0000154521.40686.63, PMID: 15781814

[ref8] Balcer L. J. Raynowska J. Nolan R. Galetta S. L. Kapoor R. Benedict R.. (2017). Validity of low-contrast letter acuity as a visual performance outcome measure for multiple sclerosis. Mult. Scler. 23, 734–747. doi: 10.1177/1352458517690822, PMID: 28206829 PMC5407511

[ref9] Balk L. J. Steenwijk M. D. Tewarie P. Daams M. Killestein J. Wattjes M. P.. (2015). Bidirectional trans-synaptic axonal degeneration in the visual pathway in multiple sclerosis. J. Neurol. Neurosurg. Psychiatry 86, 419–424. doi: 10.1136/jnnp-2014-308189, PMID: 24973342

[ref10] Behbehani R. Ahmed S. Al-Hashel J. Rousseff R. T. Alroughani R. (2017). Sensitivity of visual evoked potentials and spectral domain optical coherence tomography in early relapsing remitting multiple sclerosis. Mult. Scler. Relat. Disord. 12, 15–19. doi: 10.1016/j.msard.2016.12.005, PMID: 28283099

[ref11] Cadavid D. Balcer L. Galetta S. Aktas O. Ziemssen T. Vanopdenbosch L.. (2017). Safety and efficacy of opicinumab in acute optic neuritis (RENEW): a randomised, placebo-controlled, phase 2 trial. Lancet Neurol. 16, 189–199. doi: 10.1016/s1474-4422(16)30377-5, PMID: 28229892

[ref12] Carbajal K. S. Mironova Y. Ulrich-Lewis J. T. Kulkarni D. Grifka-Walk H. M. Huber A. K.. (2015). Th cell diversity in experimental autoimmune encephalomyelitis and multiple sclerosis. J. Immunol. 195, 2552–2559. doi: 10.4049/jimmunol.1501097, PMID: 26238492 PMC4561206

[ref13] Cordano C. Sin J. H. Timmons G. Yiu H. H. Stebbins K. Guglielmetti C.. (2022). Validating visual evoked potentials as a preclinical, quantitative biomarker for remyelination efficacy. Brain 145, 3943–3952. doi: 10.1093/brain/awac207, PMID: 35678509 PMC10200282

[ref14] Crawford D. K. Mangiardi M. Xia X. López-Valdés H. E. Tiwari-Woodruff S. K. (2009). Functional recovery of callosal axons following demyelination: a critical window. Neuroscience 164, 1407–1421. doi: 10.1016/j.neuroscience.2009.09.069, PMID: 19800949

[ref15] Cruz-Herranz A. Dietrich M. Hilla A. M. Yiu H. H. Levin M. H. Hecker C.. (2019). Monitoring retinal changes with optical coherence tomography predicts neuronal loss in experimental autoimmune encephalomyelitis. J. Neuroinflammation 16:203. doi: 10.1186/s12974-019-1583-4, PMID: 31684959 PMC6827223

[ref16] Cruz-Herranz A. Oertel F. C. Kim K. Cantó E. Timmons G. Sin J. H.. (2021). Distinctive waves of innate immune response in the retina in experimental autoimmune encephalomyelitis. JCI Insight 6:e149228. doi: 10.1172/jci.insight.149228, PMID: 34100385 PMC8262300

[ref17] Eshaghi A. Prados F. Brownlee W. J. Altmann D. R. Tur C. Cardoso M. J.. (2018). Deep gray matter volume loss drives disability worsening in multiple sclerosis. Ann. Neurol. 83, 210–222. doi: 10.1002/ana.25145, PMID: 29331092 PMC5838522

[ref18] Evonuk K. S. Doyle R. E. Moseley C. E. Thornell I. M. Adler K. Bingaman A. M.. (2020). Reduction of AMPA receptor activity on mature oligodendrocytes attenuates loss of myelinated axons in autoimmune neuroinflammation. Sci. Adv. 6:eaax5936. doi: 10.1126/sciadv.aax5936, PMID: 31934627 PMC6949032

[ref19] Feucht N. Maier M. Lepennetier G. Pettenkofer M. Wetzlmair C. Daltrozzo T.. (2019). Optical coherence tomography angiography indicates associations of the retinal vascular network and disease activity in multiple sclerosis. Mult. Scler. 25, 224–234. doi: 10.1177/1352458517750009, PMID: 29303033

[ref20] Fisher J. B. Jacobs D. A. Markowitz C. E. Galetta S. L. Volpe N. J. Nano-Schiavi M. L.. (2006). Relation of visual function to retinal nerve fiber layer thickness in multiple sclerosis. Ophthalmology 113, 324–332. doi: 10.1016/j.ophtha.2005.10.040 16406539

[ref21] Fox R. J. Cohen J. A. (2001). Multiple sclerosis: the importance of early recognition and treatment. Cleve. Clin. J. Med. 68, 157–171. doi: 10.3949/ccjm.68.2.157 11220457

[ref22] Fraser C. L. Klistorner A. Graham S. L. Garrick R. Billson F. A. Grigg J. R. (2006). Multifocal visual evoked potential analysis of inflammatory or demyelinating optic neuritis. Ophthalmology 113, 315–323.e2. doi: 10.1016/j.ophtha.2005.10.017 16406544

[ref23] Gabilondo I. Martínez-Lapiscina E. H. Martínez-Heras E. Fraga-Pumar E. Llufriu S. Ortiz S.. (2014). Trans-synaptic axonal degeneration in the visual pathway in multiple sclerosis. Ann. Neurol. 75, 98–107. doi: 10.1002/ana.24030, PMID: 24114885

[ref24] Gordon-Lipkin E. Chodkowski B. Reich D. S. Smith S. A. Pulicken M. Balcer L. J.. (2007). Retinal nerve fiber layer is associated with brain atrophy in multiple sclerosis. Neurology 69, 1603–1609. doi: 10.1212/01.wnl.0000295995.46586.ae 17938370

[ref25] Graham S. L. Klistorner A. (2017). Afferent visual pathways in multiple sclerosis: a review. Clin. Experiment. Ophthalmol. 45, 62–72. doi: 10.1111/ceo.12751, PMID: 27011293

[ref26] Graham E. C. You Y. Yiannikas C. Garrick R. Parratt J. Barnett M. H.. (2016). Progressive loss of retinal ganglion cells and axons in nonoptic neuritis eyes in multiple sclerosis: a longitudinal optical coherence tomography study. Invest. Ophthalmol. Vis. Sci. 57, 2311–2317. doi: 10.1167/iovs.15-19047, PMID: 27127930

[ref27] Graves J. Balcer L. J. (2010). Eye disorders in patients with multiple sclerosis: natural history and management. Clin. Ophthalmol. 4, 1409–1422. doi: 10.2147/opth.S6383, PMID: 21188152 PMC3000766

[ref28] Green A. J. Gelfand J. M. Cree B. A. Bevan C. Boscardin W. J. Mei F.. (2017). Clemastine fumarate as a remyelinating therapy for multiple sclerosis (ReBUILD): a randomised, controlled, double-blind, crossover trial. Lancet 390, 2481–2489. doi: 10.1016/s0140-6736(17)32346-2, PMID: 29029896

[ref29] Green A. J. McQuaid S. Hauser S. L. Allen I. V. Lyness R. (2010). Ocular pathology in multiple sclerosis: retinal atrophy and inflammation irrespective of disease duration. Brain 133, 1591–1601. doi: 10.1093/brain/awq080, PMID: 20410146 PMC2877904

[ref30] Halliday A. M. McDonald W. I. Mushin J. (1972). Delayed visual evoked response in optic neuritis. Lancet 299, 982–985. doi: 10.1016/s0140-6736(72)91155-5 4112367

[ref31] Heidari M. Radcliff A. B. McLellan G. J. Ver Hoeve J. N. Chan K. Kiland J. A.. (2019). Evoked potentials as a biomarker of remyelination. Proc. Natl. Acad. Sci. U. S. A. 116, 27074–27083. doi: 10.1073/pnas.1906358116, PMID: 31843913 PMC6936696

[ref32] Holder G. E. (1997). The pattern electroretinogram in anterior visual pathway dysfunction and its relationship to the pattern visual evoked potential: a personal clinical review of 743 eyes. Eye 11, 924–934. doi: 10.1038/eye.1997.231 9537157

[ref33] Horstmann L. Schmid H. Heinen A. P. Kurschus F. C. Dick H. B. Joachim S. C. (2013). Inflammatory demyelination induces glia alterations and ganglion cell loss in the retina of an experimental autoimmune encephalomyelitis model. J. Neuroinflammation 10:120. doi: 10.1186/1742-2094-10-120, PMID: 24090415 PMC3851328

[ref34] Huang D. Swanson E. A. Lin C. P. Schuman J. S. Stinson W. G. Chang W.. (1991). Optical coherence tomography. Science 254, 1178–1181. doi: 10.1126/science.1957169, PMID: 1957169 PMC4638169

[ref35] Ikuta F. Zimmerman H. M. (1976). Distribution of plaques in seventy autopsy cases of multiple sclerosis in the United States. Neurology 26, 26–28. doi: 10.1212/wnl.26.6_part_2.26, PMID: 944889

[ref36] Jiang H. Gameiro G. R. Liu Y. Lin Y. Hernandez J. Deng Y.. (2020). Visual function and disability are associated with increased retinal volumetric vessel density in patients with multiple sclerosis. Am. J. Ophthalmol. 213, 34–45. doi: 10.1016/j.ajo.2019.12.021, PMID: 31926161 PMC7214204

[ref37] Jin J. Smith M. D. Kersbergen C. J. Kam T. I. Viswanathan M. Martin K.. (2019). Glial pathology and retinal neurotoxicity in the anterior visual pathway in experimental autoimmune encephalomyelitis. Acta Neuropathol. Commun. 7:125. doi: 10.1186/s40478-019-0767-6, PMID: 31366377 PMC6670238

[ref38] Jones S. J. Brusa A. (2003). Neurophysiological evidence for long-term repair of MS lesions: implications for axon protection. J. Neurol. Sci. 206, 193–198. doi: 10.1016/S0022-510X(02)00428-8, PMID: 12559510

[ref39] Kallmann B. A. Fackelmann S. Toyka K. V. Rieckmann P. Reiners K. (2006). Early abnormalities of evoked potentials and future disability in patients with multiple sclerosis. Mult. Scler. J. 12, 58–65. doi: 10.1191/135248506ms1244oa, PMID: 16459720

[ref40] Kipp M. Clarner T. Dang J. Copray S. Beyer C. (2009). The cuprizone animal model: new insights into an old story. Acta Neuropathol. 118, 723–736. doi: 10.1007/s00401-009-0591-3, PMID: 19763593

[ref41] Kleerekooper I. Houston S. Dubis A. M. Trip S. A. Petzold A. (2020). Optical coherence tomography angiography (OCTA) in multiple sclerosis and Neuromyelitis Optica Spectrum disorder [review]. Front. Neurol. 11:604049. doi: 10.3389/fneur.2020.604049, PMID: 33362705 PMC7758345

[ref42] Klistorner A. Chai Y. Leocani L. Albrecht P. Aktas O. Butzkueven H.. (2018). Assessment of Opicinumab in acute optic neuritis using multifocal visual evoked potential. CNS Drugs 32, 1159–1171. doi: 10.1007/s40263-018-0575-8, PMID: 30267385 PMC6280853

[ref43] Klistorner A. Graham S. L. (2021). Role of multifocal visually evoked potential as a biomarker of demyelination, spontaneous Remyelination, and myelin repair in multiple sclerosis [review]. Front. Neurosci. 15:725187. doi: 10.3389/fnins.2021.725187, PMID: 34776840 PMC8586643

[ref44] Klistorner A. Klistorner S. You Y. Graham S. L. Yiannikas C. Parratt J.. (2022). Long-term effect of permanent demyelination on axonal survival in multiple sclerosis. Neurol Neuroimmunol Neuroinflamm 9:e1155. doi: 10.1212/nxi.0000000000001155, PMID: 35241572 PMC8893590

[ref45] Klistorner A. Triplett J. D. Barnett M. H. Yiannikas C. Barton J. Parratt J.. (2021). Latency of multifocal visual evoked potential in multiple sclerosis: a visual pathway biomarker for clinical trials of Remyelinating therapies. J. Clin. Neurophysiol. 38, 186–191. doi: 10.1097/wnp.0000000000000757, PMID: 33235179

[ref46] Lanzillo R. Cennamo G. Criscuolo C. Carotenuto A. Velotti N. Sparnelli F.. (2018). Optical coherence tomography angiography retinal vascular network assessment in multiple sclerosis. Mult. Scler. 24, 1706–1714. doi: 10.1177/1352458517729463, PMID: 28933233

[ref47] Laron M. Cheng H. Zhang B. Schiffman J. S. Tang R. A. Frishman L. J. (2010). Comparison of multifocal visual evoked potential, standard automated perimetry and optical coherence tomography in assessing visual pathway in multiple sclerosis patients. Mult. Scler. 16, 412–426. doi: 10.1177/1352458509359782, PMID: 20207786 PMC2933376

[ref48] Magon S. Tsagkas C. Gaetano L. Patel R. Naegelin Y. Amann M.. (2020). Volume loss in the deep gray matter and thalamic subnuclei: a longitudinal study on disability progression in multiple sclerosis. J. Neurol. 267, 1536–1546. doi: 10.1007/s00415-020-09740-4, PMID: 32040710

[ref49] Marenna S. Huang S. C. Dalla Costa G. d'Isa R. Castoldi V. Rossi E.. (2022). Visual evoked potentials to monitor myelin Cuprizone-induced functional changes. Front. Neurosci. 16:820155. doi: 10.3389/fnins.2022.820155, PMID: 35495042 PMC9051229

[ref50] Mey G. M. Evonuk K. S. Chappell M. K. Wolfe L. M. Singh R. Batoki J. C.. (2022a). Visual imaging as a predictor of neurodegeneration in experimental autoimmune demyelination and multiple sclerosis. Acta Neuropathol. Commun. 10:87. doi: 10.1186/s40478-022-01391-y, PMID: 35706005 PMC9199245

[ref51] Mey G. M. Mahajan K. R. DeSilva T. M. (2022b). Neurodegeneration in multiple sclerosis. WIREs Mech Dis 15:e1583. doi: 10.1002/wsbm.1583, PMID: 35948371 PMC9839517

[ref52] Murphy O. C. Kwakyi O. Iftikhar M. Zafar S. Lambe J. Pellegrini N.. (2020). Alterations in the retinal vasculature occur in multiple sclerosis and exhibit novel correlations with disability and visual function measures. Mult. Scler. 26, 815–828. doi: 10.1177/1352458519845116, PMID: 31094280 PMC6858526

[ref53] Papadopoulou A. Gaetano L. Pfister A. Altermatt A. Tsagkas C. Morency F.. (2019). Damage of the lateral geniculate nucleus in MS: assessing the missing node of the visual pathway. Neurology 92:e2240. doi: 10.1212/wnl.0000000000007450, PMID: 30971483 PMC6537126

[ref54] Paul F. Calabresi P. A. Barkhof F. Green A. J. Kardon R. Sastre-Garriga J.. (2021). Optical coherence tomography in multiple sclerosis: a 3-year prospective multicenter study. Ann. Clin. Transl. Neurol. 8, 2235–2251. doi: 10.1002/acn3.51473, PMID: 34792863 PMC8670323

[ref55] Porciatti V. (2015). Electrophysiological assessment of retinal ganglion cell function. Exp. Eye Res. 141, 164–170. doi: 10.1016/j.exer.2015.05.008, PMID: 25998495 PMC4628896

[ref56] Ransohoff R. M. (2012). Animal models of multiple sclerosis: the good, the bad and the bottom line. Nat. Neurosci. 15, 1074–1077. doi: 10.1038/nn.3168, PMID: 22837037 PMC7097342

[ref57] Ratchford J. N. Saidha S. Sotirchos E. S. Oh J. A. Seigo M. A. Eckstein C.. (2013). Active MS is associated with accelerated retinal ganglion cell/inner plexiform layer thinning. Neurology 80, 47–54. doi: 10.1212/WNL.0b013e31827b1a1c, PMID: 23267030 PMC3589201

[ref58] Rogaczewska M. Michalak S. Stopa M. (2021). Differentiation between multiple sclerosis and neuromyelitis optica spectrum disorder using optical coherence tomography angiography. Sci. Rep. 11:10697. doi: 10.1038/s41598-021-90036-6, PMID: 34021191 PMC8140093

[ref59] Saidha S. Al-Louzi O. Ratchford J. N. Bhargava P. Oh J. Newsome S. D.. (2015). Optical coherence tomography reflects brain atrophy in multiple sclerosis: a four-year study. Ann. Neurol. 78, 801–813. doi: 10.1002/ana.24487, PMID: 26190464 PMC4703093

[ref60] Schinzel J. Zimmermann H. Paul F. Ruprecht K. Hahn K. Brandt A. U.. (2014). Relations of low contrast visual acuity, quality of life and multiple sclerosis functional composite: a cross-sectional analysis. BMC Neurol. 14:31. doi: 10.1186/1471-2377-14-31, PMID: 24555757 PMC3932139

[ref61] Sekyi M. T. Lauderdale K. Atkinson K. C. Golestany B. Karim H. Feri M.. (2021). Alleviation of extensive visual pathway dysfunction by a remyelinating drug in a chronic mouse model of multiple sclerosis. Brain Pathol. 31, 312–332. doi: 10.1111/bpa.12930, PMID: 33368801 PMC8018057

[ref62] Shindler K. S. Guan Y. Ventura E. Bennett J. Rostami A. (2006). Retinal ganglion cell loss induced by acute optic neuritis in a relapsing model of multiple sclerosis. Mult. Scler. J. 12, 526–532. doi: 10.1177/1352458506070629, PMID: 17086896

[ref63] Sotirchos E. S. Gonzalez Caldito N. Filippatou A. Fitzgerald K. C. Murphy O. C. Lambe J.. (2020). Progressive multiple sclerosis is associated with faster and specific retinal layer atrophy. Ann. Neurol. 87, 885–896. doi: 10.1002/ana.25738, PMID: 32285484 PMC8682917

[ref64] Talman L. S. Bisker E. R. Sackel D. J. Long D. A. Jr. Galetta K. M. Ratchford J. N.. (2010). Longitudinal study of vision and retinal nerve fiber layer thickness in multiple sclerosis. Ann. Neurol. 67, 749–760. doi: 10.1002/ana.22005, PMID: 20517936 PMC2901775

[ref65] The Optic Neuritis Study Group (2008). Multiple sclerosis risk after optic neuritis: final optic neuritis treatment trial follow-up. Arch. Neurol. 65, 727–732. doi: 10.1001/archneur.65.6.727, PMID: 18541792 PMC2440583

[ref66] Thompson A. J. Banwell B. L. Barkhof F. Carroll W. M. Coetzee T. Comi G.. (2018). Diagnosis of multiple sclerosis: 2017 revisions of the McDonald criteria. Lancet Neurol. 17, 162–173. doi: 10.1016/S1474-4422(17)30470-2 29275977

[ref67] Tiftikcioglu B. I. Emre S. Idiman F. Idiman E. (2022). Optical coherence tomography angiography (OCTA) in differential diagnosis of aquaporin-4 antibody seronegative NMOSD and multiple sclerosis. Mult. Scler. Relat. Disord. 58:103503. doi: 10.1016/j.msard.2022.103503, PMID: 35030370

[ref68] Toussaint D. Périer O. Verstappen A. Bervoets S. (1983). Clinicopathological study of the visual pathways, eyes, and cerebral hemispheres in 32 cases of disseminated sclerosis. J. Clin. Neuroophthalmol. 3, 211–220. 6226722

[ref69] Trapp B. D. Peterson J. Ransohoff R. M. Rudick R. Mörk S. Bö L. (1998). Axonal transection in the lesions of multiple sclerosis. N. Engl. J. Med. 338, 278–285. doi: 10.1056/NEJM199801293380502 9445407

[ref70] Walter S. D. Ishikawa H. Galetta K. M. Sakai R. E. Feller D. J. Henderson S. B.. (2012). Ganglion cell loss in relation to visual disability in multiple sclerosis. Ophthalmology 119, 1250–1257. doi: 10.1016/j.ophtha.2011.11.032, PMID: 22365058 PMC3631566

[ref71] Wang L. Murphy O. Caldito N. G. Calabresi P. A. Saidha S. (2018). Emerging applications of optical coherence tomography angiography (OCTA) in neurological research. Eye Vision 5:11. doi: 10.1186/s40662-018-0104-3, PMID: 29796403 PMC5956832

[ref72] Werneburg S. Jung J. Kunjamma R. B. Ha S. K. Luciano N. J. Willis C. M.. (2020). Targeted complement inhibition at synapses prevents microglial synaptic engulfment and synapse loss in demyelinating disease. Immunity 52, 167–182.e7. doi: 10.1016/j.immuni.2019.12.004, PMID: 31883839 PMC6996144

[ref73] Wu G. F. Schwartz E. D. Lei T. Souza A. Mishra S. Jacobs D. A.. (2007). Relation of vision to global and regional brain MRI in multiple sclerosis. Neurology 69, 2128–2135. doi: 10.1212/01.wnl.0000278387.15090.5a, PMID: 17881718

[ref74] You Y. Graham E. C. Shen T. Yiannikas C. Parratt J. Gupta V.. (2018). Progressive inner nuclear layer dysfunction in non-optic neuritis eyes in MS. Neurol Neuroimmunol Neuroinflamm 5:e427. doi: 10.1212/nxi.0000000000000427, PMID: 29259999 PMC5732006

[ref75] You Y. Klistorner A. Thie J. Graham S. L. (2011). Latency delay of visual evoked potential is a real measurement of demyelination in a rat model of optic neuritis. Invest. Ophthalmol. Vis. Sci. 52, 6911–6918. doi: 10.1167/iovs.11-7434 21791585

